# Obesity May Accelerate the Aging Process

**DOI:** 10.3389/fendo.2019.00266

**Published:** 2019-05-03

**Authors:** Valentina Salvestrini, Christian Sell, Antonello Lorenzini

**Affiliations:** ^1^Department of Experimental, Diagnostic and Specialty Medicine, University of Bologna, Bologna, Italy; ^2^Department of Pathology, Drexel University College of Medicine, Philadelphia, PA, United States; ^3^Department of Biomedical and Neuromotor Sciences, Biochemistry Unit, University of Bologna, Bologna, Italy

**Keywords:** obesity, overweight, aging, aging hallmarks, caloric restriction

## Abstract

Lines of evidence from several studies have shown that increases in life expectancy are now accompanied by increased disability rate. The expanded lifespan of the aging population imposes a challenge on the continuous increase of chronic disease. The prevalence of overweight and obesity is increasing at an alarming rate in many parts of the world. Further to increasing the onset of metabolic imbalances, obesity leads to reduced life span and affects cellular and molecular processes in a fashion resembling aging. Nine key hallmarks of the aging process have been proposed. In this review, we will review these hallmarks and discuss pathophysiological changes that occur with obesity, that are similar to or contribute to those that occur during aging. We present and discuss the idea that obesity, in addition to having disease-specific effects, may accelerate the rate of aging affecting all aspects of physiology and thus shortening life span and health span.

## Introduction

The world population is aging at a rapid pace (1), we face a future where the number of elderly people will exceed that of children and there will be more people at extreme old age than ever before. The significant rise in average life expectancy during the 20th century ranks as one of society's greatest achievements. In addition, the increase in life expectancy is accompanied by a change in principal causes of disease and death, creating an “epidemiologic transition”. This transition is based on a decline in infectious and acute disease and an increase in chronic and degenerative disease (2).

The question whether living longer represents more years of healthier life or an increase in years of disability is an important question (3, 4). Evidence from several studies indicate that the recent increase in life expectancy is accompanied by an increased disability rate. The net result is no net difference in the length of a healthy life span in some estimations (5–7) and potentially a decrease in other estimates (8, 9). In a large-scale study of 187 countries examining the global burden of disease, Salomone and colleagues indicated that as life expectancy rose between 1990 and 2010, the number of healthy years lost to disability has also increased (10). This trend, which is typical of industrialized countries, has a significant impact on public health, due to the cost of care imposed by the increase in years lost to disability and underscores the need to make healthy aging a priority.

A major factor contributing to the increase in disability is an increase in obesity, creating a new and pressing challenge for public health (11–17). Obesity is expanding at a worrisome rate, the frequency of overweight and obesity combined increased by 27.5% for adults and 47.1% for children between 1980 and 2013. In developing countries, the proportion of obese adults rose from about 15% in 1980 to more than 20% in 2013 (18). Obesity is associated with an increased risk of cardiovascular disease, type 2 diabetes mellitus, cancer, osteoarthritis, work disability and sleep apnea (19).

It has been suggested that obesity not only increases the onset of metabolic imbalances, but also decreases life span and impacts cellular processes in a manner similar to aging (20).

A defining characteristic of aging is the gradual loss of physiological integrity, which results in increased vulnerability to disease and death. This loss of physiological integrity underlies multiple pathologies, including cancer, diabetes, cardiovascular disorders and neurodegenerative disease (21). Recently, nine hallmarks which define the aging process have been described (21). We will briefly discuss each of the hallmarks of aging, the potential interactions between each hallmark and obesity and, where available, the effect of caloric restriction (CR).

## Telomere Attrition

Telomeres are repetitive, non-coding, chromosomal regions located at the end of each chromosome. These telomeric regions are assembled into higher order structures, which prevent both chromosomal fusions and activation of the DNA damage response. In human somatic cells, telomere erosion occurs with each cell division creating a trigger for senescence when a critical length is reached and the telomere structure is destabilized (22).

### Aging and Telomeres

Telomere length is inversely correlated with lifespan (23), and telomere dysfunction accelerates the aging process (21). Telomere shortening, moreover, can be accelerated by factors that induce aging and attenuated by factors that improve health (24). On these grounds, telomere length has been proposed as marker of biological aging (21). Inflammation and oxidative stress have been associated with aging in general (25), and shortening of telomeres in particular.

### Obesity and Telomeres

Obesity causes oxidative stress and inflammation, which may increase the rate of telomere shortening (24). Although the association is weak or moderate, the results of a systematic review by Mundstock and colleagues show a trend toward a negative association between obesity, in particular central obesity, and telomere length (24). Human studies indicate that telomere shortening is directly correlated to adiposity (26), and telomere length is inversely associated with BMI (19). However, this association is not linear across the age and it is stronger in younger compared to older individuals (26). Interestingly, telomere length measured from subcutaneous adipocytes was significantly lower in obese patients compared to never-obese ones (27), and it appears that physical activity may protect patients from telomere shortening due to obesity, although extended periods of overweight/obesity seem to mitigate this protection (28). It should be noted that these results are not consistent in all studies (29).

Telomere length in leukocytes is linked to both obesity and smoking. For example, telomere length was inversely correlated with serum concentration of leptin, an adipokine which may contribute to an inflammatory state and elevated oxidative stress (30). Importantly for the current discussion, leukocyte telomere length, a common aging biomarker, has been shown to negatively correlate with BMI, although the association was relatively weak and gender specific (females only) (31).

### Take Home Summary

A dedicated review on the possible links between obesity, telomeres and aging concludes: “*obesity may affect telomere dynamics and accelerate the aging process*” (32). We feel that although the results cumulatively show a tendency toward an inverse correlation between obesity and telomere length; it is more prudent to conclude that the available studies are heterogeneous and show a weak statistical significance (24, 26).

## Epigenetic Alteration

Epigenetic modifications such as DNA methylation, histone modification and chromatin remodeling refer to alterations in gene expression that are inherited in descendant cells or organisms (33).

### Aging and Epigenetics

Epigenetic changes occur with age and there appears to be a relationship between epigenetic changes and age-related health problems (21). One of the strongest correlations between epigenetics and aging involves changes in a subset of methylation sites throughout the genome. These sites were identified as having an altered methylation pattern during aging and these changes have been proposed to represent an “epigenetic clock,” that may be tied to the aging process (34).

### Nutrition and Epigenetics

There is evidence that lifestyle changes, including weight loss/gain, affect gene expression by altering the DNA methylation pattern (35) and increasing the risk of developing diseases in later life. In this context, nutrition, among other environmental factors, plays a key role in inducing epigenetic changes and these changes can influence the phenotype of subsequent generations (36). Among nutrients, methyl donors play a central role. For example, folate supplementation during gestation increased DNA methylation at imprinted loci within the IGF2 gene and was associated with lower birth weight, while loss of imprinting at the IGF1 gene correlates with somatic overgrowth (36).

High fat diets (HFD) have been shown to alter the epigenome. For example, *in utero*, HFD feeding and maternal obesity alters DNA methylation patterns and histone modifications while increasing susceptibility to obesity in offspring (37). The offspring of mice exposed to a HFD exhibit modifications such as histone acetylation, in genes involved in metabolic pathways, such as glycemic homeostatic regulation. These modifications can affect the gluconeogenic capacity and potentially lead to excessive glucose production and altered insulin sensitivity in adulthood (38).

Another example of epigenetic alteration due to HFD is the increased expression of the histone deacetylase HDAC5. Increased expression of this deacetylase reduces BDNF chromatin accessibility and consequently its transcription. BDNF is a key regulator of synaptogenesis, essential for learning and memory. It has been demonstrated that HDAC5 is significantly increased in the brains of diabetic patients and in the brains of mice chronically fed a HFD (39). These epigenetic changes in the brain may persist, even after a return to normal diet, leading to pathological alterations in the cognitive machinery (40). In this study, Wang and coll. demonstrated that limited, early presence of obesity and insulin resistance may have long-term deleterious consequences in the brain, leading to a more susceptible, less resilient cognitive machinery, and contributing to the onset/progression of cognitive dysfunction, such as impairment in learning and memory formation, during aging (40).

### Obesity and Epigenetics

Besides the impact of nutrition, the direct role on aging and life span of BMI and obesity associated epigenetic changes, have also been studied. Several studies demonstrated that obesity is associated with extensive changes in gene expression in multiple tissues (41) and that increased BMI is associated with an altered methylation of specific genes (42–44). For instance, Nevalainen et al. showed that obesity is associated with methylation changes in blood leukocyte DNA that could lead to immune dysfunction. They also investigated the association between BMI and epigenetic age in blood cells and demonstrated that BMI is positively associated with epigenetic aging in middle-aged individuals (44). The impact of obesity on epigenetic aging is also described by Horvath et al. They showed that obesity accelerates epigenetic changes associated with aging in the human liver resulting in an apparent age acceleration of 2.7 years for a 10-point increase in BMI (45), supporting the idea that obesity may accelerate the aging process.

### Caloric Restriction and Epigenetics

CR as well as overnutrition can induce epigenetic alterations, which potentially impact aging. CR clearly retarded the methylation drift, thus resulting in a significantly younger “methylation age” (46). Another example is SIRT6, a stress responsive deacetylase that represents a potentially significant enzyme for aging (47, 48). Functionally, SIRT6 plays an important role in DNA repair, telomerase function, genomic stability, cellular senescence and in regulation of the transcription factor nuclear factor-κB (NF-κB), which is involved in inflammation and aging (21, 49). SIRT6 activity is significantly modulated by CR (49). Nutrient depletion or long-term CR increase SIRT6 activity in the brain, white adipose tissue, muscle, liver and kidney in mice (49, 50).

### Take Home Summary

Several reports demonstrate that nutrition and obesity are able to modulate the epigenetic signature of an individual, even during prenatal development. The observed alterations do not always overlap those seen in aging, however some studies show a close correlation between epigenetic alteration induced by obesity and an acceleration of tissue aging. This suggests that obesity could accelerate age-related dysfunction by inducing epigenetic alterations that are not necessarily the same as those observed during aging in non-obese individuals.

## Mitochondrial Dysfunction

Mitochondria play a central role in bioenergetic metabolism and ATP production, and maintenance of their function across lifespan is essential for general homeostasis.

### Aging and Mitochondria

Because of their key role in multiple cellular functions, these organelles are involved in multiple distinct processes with relation to aging, including: inflammation, mitophagy and proteolysis, the mitochondrial unfolded protein response, cellular senescence, stem cell function, accumulation of DNA mutations, and bioenergetics alterations [reviewed in (51)]. During the aging process, a reduction in the efficiency of mitochondrial bioenergetics has been observed and several involved mechanisms have been described, such as a reduced biogenesis, mutation in mtDNA, alteration in mitochondrial dynamics (imbalance fission/fusion) or defective mitophagy (52).

Both excessive nutrient consumption and obesity have been linked with mitochondrial dysfunctions.

### Excessive Nutrient Consumption and Mitochondria

During excessive nutrient consumption, the metabolism shifts toward increased lipid storage and glycolytic ATP synthesis while concurrently decreasing mitochondrial biogenesis (53). Mitochondria play an essential role in nutrient adaptation; excessive consumption of nutrients affects their functions in those tissues that participate to nutrient metabolism: adipose tissue, liver, and skeletal muscle. Excessive nutrient intake also increases the concentration of free fatty acids and mitochondrial ROS production, leading to hyperglycemia and adipocyte mitochondrial dysfunction. In this tissue, mitochondrial biogenesis, mtDNA content and the rate of β-oxidation are reduced while adipogenesis, fatty acids esterification and lipolysis are altered. These modifications contribute to alteration of insulin sensitivity (54).

### Obesity and Mitochondria

Obesity has also been associated with mitochondrial dysfunctions (54). CR, conversely, which increases longevity, maintains mitochondrial function (55). Several studies showed that obesity induces a reduction in mitochondrial biogenesis and a decreased mitochondrial oxidative capacity in adipocytes of both rodents (56) and humans (54). In obese individuals, reduced mitochondrial biogenesis is associated with metabolic alterations, low-grade inflammation, and insulin resistance (57). Several lines of evidence (54) suggest that obesity induces a shift toward a fission process linked to mitochondrial dysfunction in liver and skeletal muscle. In skeletal muscle of obese mice, an increased mitochondrial fission was observed and the activity of protein involved in mitochondrial dynamic was altered (58).

Some of the adaptations observed under excessive nutrient consumption are also observed in obesity; mitochondria of obese individuals show a reduced oxidation of fatty acids, have less defined internal membranes, a lower energy generation capacity and an increased glucose dependence for ATP synthesis (59).

Mitochondria are also a central players in apoptosis (60), and the availability or ingestion of nutrients is related to the regulation of cell death. Excessive food intake impairs mitochondrial respiratory capacity and sensitizes mitochondria to apoptotic stimuli (59). Obesity upregulates apoptotic pathways proteins in rodent and humans, and increased apoptosis in adipocytes, as demonstrated by an association between body fat and a pro-apoptotic state in adipose tissue of obese patients (54).

### Take Home Summary

Mitochondrial dysfunction occurs in aged tissues, in response to excessive nutrient intake, and in obesity, contributing to inflammation and insulin resistance. Aging and obesity appear superimposable in their impact on mitochondria and it is reasonable to hypothesize that they could exert additive effects.

## Cellular Senescence

Cellular senescence is an irreversible block of the cell cycle that limits the proliferative potential of cells (61). Cellular senescence, along with apoptosis, is a physiological process that plays a crucial role in the removal of damaged cells and tissue remodeling; it is a crucial mechanism for development but becomes deleterious when it affects stem and immune cell function, impacting tissue homeostasis. Senescence can be triggered by several stress stimuli, such as telomere uncapping, DNA damage and oncogene activation (62). Senescent cells have a large flattened morphology, stop DNA replication, show increased levels of proteins involved in cell cycle arrest and tumor suppression (such as the tumor suppressor p53 and cyclin-dependent kinase inhibitors [CDKi] p16^INK4A^, p21^CIP1/WAF1^, and p15^INK4B^), and are positive for the senescence-associated β-galactosidase (SA β-gal). They also display altered histone modification profiles and an altered secretome consisting of pro-inflammatory factors, growth factors and proteases, the so called senescence associate secretory phenotype: SASP (63). SASP factors influence the behavior of neighboring cells, resulting in the paracrine induction of senescence, tissue remodeling, and recruitment of immune cells (e.g., T lymphocyte and macrophage) (63). Although senescent cells are resistant to apoptosis, their activation of immune system causes removal of nearby cells as well as the senescent cells themselves (64).

### Aging and Cellular Senescence

During the initial description of replicative senescence, Hayflick proposed that the process may contribute to organismal aging (65). Although two key axioms of this idea, i.e., the relationship of replicative senescence with donor age or with species longevity are not supported by subsequent experiments [reviewed in (66)], an increase in senescent cells has been observed *in vivo* in different tissues (67, 68). Perhaps more importantly, the clearance of accumulated senescent cells in tissue during aging has been demonstrated to extend median lifespan and to attenuate age-related deterioration of organs in mice (69).

### Obesity and Cellular Senescence

It has been demonstrated that SA β-gal^+^ cells are more abundant in pre-adipocyte and endothelial cells isolated from obese compared to lean rats and human, moreover there is a positive correlation between BMI and adipose tissue SA β-gal activity and p53 [reviewed in (64)]. There is an accumulation of senescent T cells and an increased number of macrophages in the inflammatory foci of the visceral adipose tissue of HFD-fed obese mice (70), and obese mice accumulate senescent glial cells in the brain (71).

### Adipocyte Cellular Senescence

Senescent pre-adipocytes are defective in their differentiation capacity; it has been shown that senescent adipose-derived stromal/progenitor cells express reduced levels of adipogenic regulators and altered expression of adipogenic differentiation gene patterns in response to adipogenic hormone stimuli (72). In the heterochronic parabiosis model, blood from 3 months old mice is able to reduce the levels of pro-inflammatory cytokines in the visceral adipose tissue of 18 months old mice (73).

### Take Home Summary

There appears to be a strong relationship between obesity and senescence. Reports like the ones described above suggest that obesity may promote the aging process by inducing senescence. Conversely, senescence and the resulting pro-inflammatory secretory phenotype could contribute to the morbidity associated with obesity and plays a role in the development of insulin resistance and diabetes. There is vast literature in support of this view, and we refer the interested readers to gather valuable in depth reviews (74–76). Finally, Fontana et al. have proposed that CR might exert its anti-aging capacities by limiting senescent cell accumulation (77).

## Stem Cell Exhaustion

### Aging and Stem Cells

There is increasing evidence that the aging process can have adverse effects on stem cells. As stem cells age, their renewal ability deteriorates and their ability to differentiate into the various cell types is altered (78). The life-long persistence of stem cells in the body makes them particularly susceptible to the accumulation of cellular damage, which ultimately can lead to cell death, senescence or loss of regenerative function (79). These changes translate into reduced effectiveness of cell replacement and tissue regeneration in aged organisms.

### Obesity and Stem Cells

Obesity is associated with a pro-inflammatory response in a wide variety of tissues. Inflammation can activate the stem cell compartment with negative consequences. For example, a reduction in functionally active stem cells has been observed in subcutaneous adipose tissue from obese patients (80). Adipose-derived stem cells (ASC) isolated from obese patients demonstrated a reduced proliferative ability and a loss of viability together with changes in telomerase activity and telomere length (81). Moreover, their mitochondrial content and function are altered. Specifically, ASC contain a greater number of mitochondria and produce more ROS, however their mitochondria show a reduced respiration capacity, concomitant with a shift toward β-oxidation instead of glycolysis for energy production.

ASC from obese patients have reduced differentiation potential and are less proangiogenic (80), which is reflected in differences in their gene expression profile (82). Onate and colleague, demonstrated that obesity impairs the expression of genes involved in regulation of cell proliferation, differentiation and angiogenic potential of ASC, rendering them less multipotent. Moreover, obesity seems to affect ASC trafficking and homing (82), changes which may reduce the capacity of these cells for tissue repair (80).

Obesity also influences bone marrow (BM) homeostasis, increasing adipocyte formation. Bone marrow adipose tissue (BMAT) originates from bone marrow stromal stem cells (BM-MSC), which give rise to adipocyte, osteoblast and hematopoietic-supporting stroma (83). BMAT is an endocrine-active fat depot capable of influencing BM stem cells. Chronic low-grade inflammation associated with obesity is a stressor for BM stem cells due to the continuous response to inflammatory cytokines. In turn, inflammation causes alterations in the microenvironment with implications for cell production. In mice, HFD-induced obesity leads to a progenitor cell exhaustion and impairs osteoblast recruitment and bone formation, decreasing proliferative potential of progenitor cells and enhancing adipocytic differentiation of BM-MSC (83).

Obesity has a direct effect on the hematopoietic stem cell (HSC) compartment; however, obesity and aging seem to have different effects. HSC aging leads to a paradoxical increase in the stem cell pool and decline in stem cell function. One of the prominent modifications of HSC properties with age is their biased differentiation toward myeloid lineage at the expense of their lymphoid potential (84). None of these characteristics are observed in obesity. An elegant study by Lee et al. demonstrated that obesity leads to changes in the cellular architecture of the stem cell compartment. HSCs acquire an immature phenotype, remain quiescent, and are refractory to the low-grade inflammation signals, while differentiated progenitors are more greatly affected. Through the use of a genetic mouse model, the authors demonstrated that obesity affects the long-term reconstitution ability of HSC while also leading to an exacerbated proliferative response of multipotent progenitors. These effects are linked to the upregulation of Gfi1, a key regulator of HSC quiescence and self-renewal, in response to the oxidative stress associated with obesity (85). The aberrant HSCs activity is progressively acquired during weight gain but it is long lasting after weight loss, demonstrating that obesity induces lasting changes in the HSC compartment (85).

It has been demonstrated that postnatal overnutrition reduces myogenic stem cell frequency and function (86) and that HFD fed mice show a reduced number of neural stem cells in the hypothalamus with a reduced differentiation capacity (87).

### Caloric Restriction and Stem Cells

Stem cells are adapting their metabolism in response to environmental changes, they skew toward a quiescent state in case of stress, or begin to proliferate-differentiate in response to injury (88). The effects of diet on stem cell metabolism and function have been assessed in response to CR. CR slows down age-related decline and enhances stem cell activity by altering their metabolic activity, promoting oxidative phosphorylation over glycolysis (88). CR potentially shifts the balance toward self-renewal while reducing the numbers of differentiated cells, thus preserving the stem cell pool and preventing stem cell exhaustion (89). In both young and old mice, CR increases the frequency and function of skeletal muscle stem cells by increasing mitochondrial content and promoting oxidative metabolism (90).

### Take Home Summary

With the exception represented by the effects on the HSC compartment, both obesity and aging, negatively impact ASC, neural stem cells and BM homeostasis. In contrast, CR promotes self-renewal and prevents stem cell exhaustion. Overall, obesity does not mimic aging in terms of stem cells compartments but, similar to aging, has a disrupting influence on their tissue maintenance functions.

## Deregulated Nutrient Sensing

The major signal pathways that participate in nutrient sensing are: the insulin/ insulin-like growth factor (IGF-1) signaling (IIS) pathway which informs the cell of the presence of glucose (and IGF-1); mTOR, for sensing amino acid concentrations (and integrating this information with growth factor signals from the IIS); AMPK which senses low-energy state by detecting low level of ATP; and sirtuins which sense nutrient scarcity by detecting high NAD+ levels.

### Aging and Nutrient Sensing

Trophic signals that activate IIS or the mTOR pathways are now considered major accelerators of aging. Multiple studies in mutant mice show that a reduction in the growth hormone (GH)/ IGF-1 signaling extends life span [reviewed in (91)] and at least one study points to an IGF-1 independent role of GH (92). mTOR activity is now regarded by many as a central player in dictating the pace of aging (93). On the opposite side, upregulation of the AMPK and sirtuins pathways may mediate lifespan extension (21).

### Obesity and the Insulin/ IGF-1 Signaling (IIS) Pathway

Mediators of inflammatory signals such as c-Jun NH2-terminal kinase (JNK) and kB kinase-B (IKKβ) impair insulin signal pathways, in turn interfering with the phosphorylation of receptor substrate 1 (IRS1), reducing the interaction with PI3K and consequently reducing glucose uptake (94). In obesity, altered insulin action and the consequental PI3K/Akt signaling pathway alteration in skeletal muscle, liver and adipose tissue may cause systemic insulin resistance (95). In skeletal muscle, insulin resistance leads to a decreased glucose transport, and a reduction in glycogen synthesis. In liver, insulin resistance results in a failure of gluconeogenesis suppression, however it stimulates fatty acid synthesis. Adipose tissue shows altered insulin-stimulated glucose transport and lipolysis. However, not all insulin signaling is diminished. For example, in liver tissue, the gluconeogenic pathway becomes insulin resistant, although insulin dependent lipogenesis stays sensitive (96).

When caloric restriction is present, the liver produces less IGF-1 and it is refractory to GH stimulation (97). While it has been clearly demonstrated that hyperinsulinaemia in obesity leads to significantly reduced GH secretion, which affects insulin's ability to maintain normal glucose homeostasis (98). The effects of obesity on IGF-1 levels are more controversial. Chronic hyperinsulinemia is associated with increased circulating IGF-1 levels. Insulin suppresses IGF binding protein (IGFBP)-1 and -2, which reduce the bioavailability of IGF-1 in the peripheral tissues (98). Increasing BMI is associated with a reduction of IGFBP-1 and IGFBP-2 expression and consequently with high circulating free IGF-1 levels (98). However, while it has been reported that IGF-1 levels are high in obesity, other studies show that it is not increased or may even be decreased (98). These differences may be due to methodological challenges associated with IGF-1 measurements.

Diet and surgical induced weight loss can revert the defects in the GH/IGF-I axis in obesity (99).

### Obesity and the mTOR Pathway

Obesity promotes mTOR activity in adipose tissue, leading to exacerbated hyperlipidemia and insulin resistance (100). For example, mTORC1 is hyperactivated in tissue of obese and HFD fed rodents (101) and genetic variation in Raptor, an mTOR-interacting partner, is associated with overweight/obesity in American men of Japanese ancestry (102). High adiposity is closely associated with development of insulin resistance, and it has been demonstrated that in the state of overnutrition, one of the molecular factors involved in insulin resistance is the ribosomal protein S6 kinase 1 (S6K1), a downstream target of mTOR signaling (103).

Decreased activation of mTOR/S6K1 has been associated with increased insulin sensitivity (104). S6K1 is hyperactivated in the adipose tissue, liver and muscle of different genetic mouse model of obesity. It has been described that HFD fed *s6k1* deficient mice are protected from developing obesity and insulin resistance (103). Chronic activation of the mTOR/S6K1 pathway by insulin, TNF-α and amino acids promote insulin resistance in obese mice and primary cultures of skeletal muscle cells from patients with type 2 diabetes through increased IRS1 serine phosphorylation and degradation (105). The HFD fed *s6k1* deficient mice show a strong reduction of phosphorylation of these sites; suggesting that S6K1 inhibits insulin signaling by mediating IRS1 phosphorylation (104). Moreover, obese patients express increased levels of *RPS6KB1*, the human gene encoding S6K1, in visceral fat compared to lean volunteers (103). Fat mass reduction after CR is associated with adipose tissue mTOR inhibition. Accordingly, pharmacological inhibition of mTORC1 pathway is associated with a reduction of both adipocyte size and number (106). Deletion of the mTORC1 target p70S6K protects against age- and diet-induced obesity (104).

### Obesity and the AMPK Pathway

Obesity induces a broad, non-tissue, or isoform specific decrease in AMPK activity (107). HFD substantially inhibits AMPK activity in white adipose tissue, heart and liver, and this reduced activity is associated with systemic insulin resistance and hyperlipidemia (107). It has been reported that AMPK activity is lower in morbidly obese humans who are insulin resistant than in comparably obese individuals who are insulin sensitive (108). AMPK activity is also reduced in the paraventricular nucleus of mice with diet-induced obesity (109). In addition, some studies demonstrated the impairment of AMPK activation in skeletal muscle of individuals with obesity and diabetes (110, 111), and in visceral adipose tissue of centrally obese humans with Cushings syndrome, a disorder associated with insulin resistance (112).

### Obesity and Sirtuins

There is a correlation between obesity and reduced Sirt1 levels. Adipose tissue from HFD fed mice (113) and db/db leptin resistant obese mice (114) show a significant reduction of Sirt1. Lower levels of Sirt1 have been reported in obese pigs compared to lean ones (115), and Choi et al. demonstrated that microRNA mir34a, which is elevated in obesity, reduces NAD+ levels and Sirt1 activity (116). Observational studies demonstrated the association between changes in sirtuins and obesity in human. Reduced mRNA levels of Sirt1 were observed in adipose tissue from obese women compared to lean women (117) and in peripheral blood mononuclear cells of diabetic subjects with insulin resistance (118). Furthermore, an increased expression of Sirt1 and Sirt3 was observed in adipose tissue of severely obese patients who experienced weight loss after gastric banding surgery (119).

### Take Home Summary

In biogerontology, the IIS and mTOR pathway are considered “accelerators” of the aging process. There is accumulating literature suggesting that in obesity, these pathways are over-activated. In contrast, there is also accumulating literature showing that pro longevity pathways, such as the AMPK and sirtuins pathways are dampened by obesity. In conclusion, there is solid evidence that obesity deregulates cellular mechanisms related to nutrient sensing.

## Altered Intercellular Communication

It is accepted that aging impacts the organism at the cellular level, but also decreases the capacity of cells of an organism to interact.

### Aging and Intercellular Communication

During aging, there is a decreased communication at the neuronal, neuroendocrine and endocrine levels. Two of the most compelling examples of impaired communication are inflammaging and immunosenescence (120). Inflammaging refers to the concept that aging is accompanied by a proinflammatory state, which is the consequence of multiple conditions, SASP, defects in autophagy and mitophagy, an enhanced activation of the inflammatory mediator, NF-κB. This phenotype results in elevated cytokines such as: IL-1b, tumor necrosis factor, and interferons. These cytokines can accelerate and propagate the aging process. Immunosenescence refers to the decreasing efficiency of the adaptive immune system with aging.

### Obesity and Pro-inflammatory Cytokines

With obesity, the adipocyte secretome changes toward greater secretion of pro-inflammatory mediators and reduced production of anti-inflammatory or insulin sensitizing factors (121). More precisely, hypertrophic conditions induce adipocyte stress, activating Jun N-terminal kinase (JNK), NF-κB, Ask1, and MKK4. Activation of these pathways induce adipocytes, endothelial cells and immune cells to produce pro-inflammatory cytokines, endothelial adhesion molecules, proatherogenic and chemotactic mediators [IL-6, tumor necrosis factor- α (TNF-α), IL-1β, MCP-1, PAI-1, Csf-1, progranulin, chemerin, and others] in adipose tissue (122). These changes impact both number and function of immune cells, increasing the number and the activity of a subset (macrophages, neutrophils, mast cells, B, and T lymphocytes) and other subtypes [eosinophils, T helper 2, Treg and natural killer T cells (NKT)] (123, 124).

It has been demonstrated that macrophage number increases with adiposity, and the accumulation is greatest in visceral fat in humans (124). Chemoattractant molecules, such as MCP-1, secreted by adipocytes, recruit monocytes from peripheral blood to adipose tissue, where they differentiate into macrophages (125). Moreover, MCP-1 promotes the local proliferation of adipose-resident macrophages. Monocytes migrate in adipose tissue in response to adipocyte-derived cell stress markers, including CCL5, IL-6, IFN-γ and TNF-α which enhance macrophage accumulation and their polarization toward a pro-inflammatory M2 phenotype (124). The increased number of macrophages has a positive correlation with the degree of insulin resistance in both mice and humans (124). Elevated chemokine ligand (CXCL)-2 release by adipose tissue promotes neutrophil infiltration, which are 20-fold more abundant in adipose tissue from HFD fed mice compared to chow-fed ones (124). On the contrary, obesity decreases AT eosinophil numbers leading to reduced insulin sensitivity while an increase in eosinophils in response to IL-15 overexpression improves obesity-induced insulin resistance (123).

CD4+ Th1 cells increase in human subcutaneous adipose tissue with obesity and exhibit an activated CD25+ phenotype. HFD fed mice show an increased IFN- γ secretion, that impaired insulin signaling and promoted macrophage infiltration, as a consequence of Th1 cell predominance (124). As with CD4+, obesity also increases CD8+ T cell levels along with their products, granzyme B and IFN-γ. In obesity, Treg cells, suppressors of inflammatory reactions, are decreased both in their proliferative capacity and in number. Dendritic cells accumulate in AT of HFD fed mice, and induce a pro-inflammatory microenvironment by secreting IL-6 and promoting macrophage recruitment/proliferation, following enhanced INF signaling and MCP-1 production (124).

The resulting imbalance in immunological phenotypes leads to development of local inflammation that further spreads into systemic circulation affecting other organs. For example, the adipokine Haptoglobin (Hp), a clinical marker of inflammation increased in the cerebral spinal fluid of patients with neurodegenerative disorders, has an abundance positively related to body fat in adipose tissue and plasma (126, 127).

Because inflammaging contributes to immunosenescence, the obesity-derived inflammatory status reduces the efficiency of the immune system, consistent with the observation that obese people are more susceptible to infection from bacteria, fungi or viruses [see an article part of this Research Topic, (128)]. For a more in depth discussion of the interconnections between adipokines and aging we refer the reader to two additional articles which are part of this Research Topic (129, 130).

### Obesity and Extracellular Vesicles

Extracellular vesicles (EV, micro-vesicle and exosomes) are nanoparticles that contain protein and nucleic acids, which interact with target tissues. Exosomes are increased in many inflammatory conditions (131), and increased numbers of microvescicles have been associated with obesity (132), while a significant reduction occurs in CR or following bariatric surgery in obese patients (133).

Several studies have demonstrated that EVs collected from adipose tissue of *ob/ob* obese mice induce, in a target cell population, changes consistent with the obese phenotype (134). EV treated monocytes were more activated, secreted more IL-6 and TNF-α compared to those treated with EV from wild type mice, and macrophages were more activated and had an increased homing capacity to adipose tissue and liver (134). In humans, EVs isolated from adipose tissue induced monocytes to adopt properties characteristic of adipose tissue macrophages (135).

Finally, it has been demonstrated that obesity reduces the pro-angiogenic potential of adipose tissue stem cell-derived EV by reducing VEGF, MMP-2 and miR-126 content (136).

### Take Home Summary

The literature persuasively suggests that the accumulation of pro-inflammatory cells, in the adipose tissue of obese patients, through cytokines and extracellular vesicles, accelerates the rate of aging both in the adipose tissue itself and the entire organism.

## Genomic Instability

### Aging and Genomic Instability

The hypothesis that aging may result from the accumulation of DNA damage is one of the classical theories of aging (137), and is supported by considerable evidence. For example, accumulation of DNA damage (138) and mutations (139) with increasing chronological age. Longevity differs by several orders of magnitude among animals and long life spans seem to associate with a greater capacity to detect the presence of DNA damage at the cellular level [reviewed in (140)]. Enhanced recognition of damage should allow enhanced DNA repair. In mammals, there is an exponential relationship between longevity and the capacity to perform the first step of non-homologous end-joining, i.e., the recognition of linear DNA ends (141), resulting in improved genomic stability (140, 142).

### Obesity and Genomic Instability

The impact of obesity on genomic instability has been analyzed in a recent review by Setayesh et al. (143). Results from animal studies and from 39 studies in humans, monitoring DNA damage in lymphocytes and sperm, were analyzed. However, heterogeneity in the study design, methodology, and confounding factors, preclude the conclusion that an association exists between obesity and DNA damage. Nevertheless, the causal relation between excess of body weight and genomic instability is supported by mechanistic studies.

Several molecular mechanism may cause genetic instability in overweight/obese individuals; one of them is oxidative stress. Oxygen derived free radicals may act as potential cytotoxic intermediates inducing inflammatory and degenerative processes, or as signal messengers for the regulation of gene expression. Many articles show evidence for the induction of oxidative DNA damage and a decreased antioxidant capacity in obesity. High glucose levels directly, and high insulin levels, through the over activation of its signaling pathways, could be responsible for increased ROS formation. Othman et al. demonstrated that insulin causes DNA damage in kidney cells (144); it has been demonstrated that insulin and glucose blood level correlate with DNA damage in Korean men (145), as well as DNA damage in sperm in a mouse model (146). Other studies provide evidence for a reduction of antioxidant enzymes and a subsequent oxidative stress as a consequence of obesity, in human and mice (143).

Oxidative stress leads to oxidation of fatty acids, and some metabolites of lipid peroxidation are molecules that attack DNA and are involved in the etiology of cancer. Elevated levels of lipid peroxidation markers are observed in blood, muscles and adipose tissue of obese individuals. Excess body weight causes hormonal imbalance and it has been demonstrated that alterations of hormonal status plays a role in some cancers, including breast and endometrial cancer (143). Hormones increase the mitotic activity of breast cells, leading to accumulation of errors in DNA replication that are frequently converted in persistent mutations (147). Some products of estrogen metabolism cause direct DNA damage. Metabolites of estradiol are mutagenic in rat and human cells, and the association between genotoxic estrogen metabolites and breast cancer is well documented (143). Another mechanism by which ROS are generated is glycation. Glycation end products cause DNA damage directly and *via* interaction with signaling pathways. They bind specific receptors and activate NADH-oxidase to induce ROS formation (148). Elevated concentrations of glycation end products have been observed in adipose tissue and the livers of HFD fed mice (149), as well as human adipose tissue (150). In patients with metabolic syndrome, elevated serum levels of glycation end products correlate with markers of insulin resistance and inflammation (151).

The persistent production of ROS by inflammatory cells present in adipose tissue damages macromolecules (DNA, RNA, lipids, carbohydrates and proteins), induces genomic instability and tips the balance from an antitumor activity of ROS to a tumor promoting one (152).

Obesity is strongly associated with an increased incidence of cancer both in humans (153) and in rodents (143). An impact of obesity has been described also on infertility, and some studies (154, 155) demonstrated an increase in DNA damage in the sperm of obese men.

Obesity, moreover, impacts the DNA repair process. For example, the offspring of mice fed a low folate diet showed a reduced base excision repair capacity in several brain regions when exposed to HFD (156). There is an inverse association between adiposity and nucleotide excision repair (157), while some studies demonstrate an altered methylation pattern of genes involved in DNA repair in overweight individuals (143). Significantly, HFD reduces the expression of MLH1, a protein involved in DNA mismatch repair, and elevate CpG methylation in mice (158).

### Caloric Restriction and Genomic Instability

Several studies demonstrated a beneficial impact of CR on genomic stability. CR slows down the rate of DNA damage by decreasing the levels of oxidative stress and by increasing the expression of stress response genes to enhance DNA repair (159). Although the vast majority of available studies are on rodents, there is also evidence in humans; for example, after 12 months of bariatric surgery, using the comet assay, a significant reduction in DNA damage is observed in peripheral blood cells (160).

### Take Home Summary

Although much research has been performed, the assumption of a relation between obesity and genomic instability is not supported unequivocally. Oxidative damage seems as the one mechanism regarded as the most relevant (161).

## Loss Of Proteostasis

Proteins represent a key components of cells and tissues, and protein quality control and homeostasis are critical to the organism. Proteostasis is maintained through multiple mechanisms ensuring stabilization of correctly folded proteins and degradation of misfolded proteins. The first task is performed by chaperones, most prominently the heat shock protein (HSP) family, and the second task is performed by proteasome and lysosome mediated degradation (162). Proteins are synthetized in the endoplasmic reticulum (ER) where unfolded proteins are bound by the ER stress sensor Binding immunoglobulin protein (BiP/GRP78) to trigger the unfolded protein response (UPR). The existence of this evolutionarily conserved mechanism provides evidence of the critical role of proteostasis.

### Aging and Loss of Proteostasis

With age, the ability of many cells and organs to preserve proteostasis under resting and stressful conditions is gradually compromised (162). Key pathways affected by the aging process alter components of the proteostasis machinery, e.g., by inducing reduction of chaperones or proteasomal degradation (163, 164). The consequent increase in misfolded or degraded proteins can lead do the development of age-related pathologies such as Alzheimer and Parkinson diseases (165).

### Obesity and Loss of Proteostasis

Obesity can induce prolonged or chronic UPR response (166) possibly mediated by proteasome dysfunctions (167). In the livers of mouse models of obesity and in HFD fed mice, proteasome activity is reduced and polyubiquinated proteins accumulate. In these mice, impaired proteasome function leads to hepatic steatosis, hepatic insulin resistance, and UPR activation. Treatment with chemical chaperones partially reverted this phenotype (168). Elevated free fatty acids in obesity activate the UPR in both adipose tissue and liver (167). It has been demonstrated that ER protein folding is impaired in the liver and within adipose tissue of obese mice. Overexpression of the chaperone BiP/GRP78 in the liver of *ob/ob* mice reduces UPR activation markers, hepatic steatosis, and improved insulin action (169). Obesity-induced changes in ER calcium store can induce a reduction in chaperone-mediated protein folding activity, ER stress and UPR activation (167). Cholesterol and free fatty acids induce ER stress via increased reactive oxygen species, and ER Ca^2+^depletion from sarco/endoplasmic reticulum calcium ATPase (SERCA) dysfunction. Diminished SERCA expression and activity were observed in livers and macrophages of obese and insulin resistant mice, which also have higher level of ER stress (170). Overexpression of SERCA in obese mice reduced UPR activation and improved glucose homeostasis (167).

Other key regulators of ER homeostasis are the three luminal sensor inositole, requiring protein 1 (IRE1α), protein kinase RNA-like ER kinase (PERK) and activating transcription factor 6 (ATF6). Obesity leads to a disproportionate production of these key molecules. A reduction of ATF6 in the presence of a sustained PERK activation was observed (171). Hyperactivation of the IRE1α-XBP1 pathway has been documented in the adipose tissue of obese human (172). Shan *et al*. found that IRE1α was activated in adipose tissue macrophages and adipocytes of HFD fed mice (172). Their observations are consistent with other studies showing that in genetic and diet-induced models of obesity, IRE1α undergoes prominent activation (173).

A link between HSPs and obesity was suggested by observations that HSP70 was reduced in muscles of insulin-resistance obese patients with type 2 diabetes (174). Subsequently, several studies confirmed an altered expression of HSPs in obese humans and animals (94). Binding of extracellular HSP60 to TLR4 triggers a proinflammatory response and promotes insulin sensitivity (175). Obese subjects with or without type 2 diabetes have high HSP60 plasma levels. Interestingly, a reduced expression was observed in morbidly obese individuals after weight loss due to bariatric surgery (94).

Upregulation of HSP72 and HSP25 was previously shown to inhibit JNK and IKK-β activation, improve glucose tolerance, restore insulin-stimulated glucose transport, and increase insulin signaling in skeletal muscles from rats fed at high-fat diet (176).

Misfolded proteins can also be removed through autophagy, which is enhanced by CR (177). Autophagy is regulated by the integrated action of insulin and mTOR, both altered in obesity (178). Bugliani et al. have recently reported that promotion of autophagy increases survival of human pancreatic beta cells under ER stress and in type 2 diabetes (179). Yang et al. demonstrated that defective autophagy is causal to impaired hepatic insulin sensitivity and glucose homeostasis (178). Persistent IIS signaling in cell culture decreases autophagy and cell viability (164). Significant autophagy defects are observed related to ER stress in the liver tissue of HFD fed mice, and restoration of autophagy reduced ER stress (178).

### Take Home Summary

Here we have briefly reviewed the abundant literature indicating that obesity significantly decreases mechanisms associated with proteome maintenance.

## Conclusions

### Two, Not Mutually Exclusive, Hypotheses

**Figure 1:**
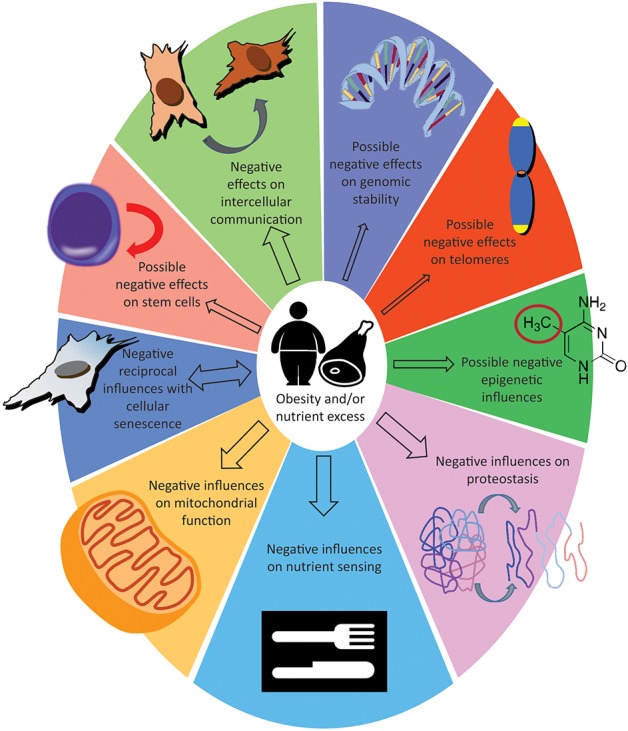
Effect of obesity or nutrient excess on the hallmarks of aging. The size of the arrows indicate how solid are the evidences, see “take home summaries” in the text. The double-headed arrow for cellular senescence indicate that detrimental influences can feedback from senescence to obesity.

We have reviewed and organized the literature with the intent of showing the existing parallels between excessive fat accumulation and the aging process. We have categorized these reports following what have been proposed to be the nine hallmarks of aging (21) (Figure 1). Based on the evidence, two distinct hypotheses can be proposed. One is that the cellular responses provoked by an excess of nutrients cause obesity, and that obesity is responsible for accelerating the pace of aging. Supporting this hypothesis are the observations that knocking out the fat-specific insulin receptor, to produce extremely lean mice (180), and removal of visceral fat in rats (181) increased life span; additionally, CR on lean strains of rats, had only a minor effects on lifespan (182, 183). The alternative possibility is that the cellular responses provoked by an excess of nutrients are responsible for increasing the pace of aging. This common soil shared by both aging and obesity has been named “adipaging” (184), and there is some evidence of commonalities: hyperglycaemia, for example, induces senescence and the SASP in endothelial cells and macrophages (185) while glucose reduction prevents replicative senescence in human mesenchymal stem cells (186). The more abundant macronutrients (by weight and by calories) in the diet are usually carbohydrates and lipids, and specific reviews are available that focus on the possible toxic effects of their respective excess: carbotoxicity (187) and lipotoxicity (188). In this second scenario, obesity represents only a side effect of the excess nutrient status and the fulcrum are the cellular nutrient sensing pathways; see for example the possible central role of mTOR (189, 190). Whether adipose tissue hyper-function/dysfunction is causative of aging functional decline or whether it represents simply a marker of the advancing aging process will become clearer with future studies. In addition, not all fat depots are equal in their impact to health (191) and it could also turn out that both hypotheses are concurrently true (192).

### Will Caloric Restriction Work on Humans?

While large epidemiological analyses indicate obesity will threaten any future gains in life expectancy (193), caloric restriction is considered a powerful tool to slow down the pace of aging. The effect of CR on increasing life span were first observed in rodents in 1935 (194). Although there are important differences between mouse, rat and human fat depots (195), the CR regiment has a clear impact in reducing fat mass in all these species (196). Caloric restriction studies are undoubtedly highly valuable for understanding the aging process; however, how to translate the data on animal models to human is a highly debated issue (197–204).

One caveat is that the response of our species could not be as strong as the one observed in experimental species. In fact, although the extension of life span with CR seems a universal biological response, this response is particularly evident in model species (*Saccharomyces cerevisiae, Caenorhabditis elegans, Drosophila melanogaster* and Rodents) suggesting involuntary selection caused by human husbandry (205). Additionally, while wild-*Drosophila* respond to CR (206), wild-mice do not (207).

Moreover, it is not clear if all humans will benefit from CR. Data on diets mimicking fasting (208), a more recent approach to caloric restriction, suggest that this approach is effective, particularly on subjects at risk for metabolic disease (209). Additionally, while extreme obesity consistently increases all-cause mortality, overweight and even mild obesity appears protective toward cardiovascular disease. A phenomenon well described and referred to as the obesity paradox (210). This stresses the importance of carefully considering the starting BMI level before suggesting CR to people. Lorenzini has proposed that initial BMI levels could be the main reason why the two larger CR studies on rhesus monkeys gave discordant result on longevity (201).

If the possibilities raised in this review are correct, we will have to conclude that it is not CR that is slowing down aging but it is the *ad libitum* feeding, coupled with the lack of physical activity (the typical condition of laboratory animals) that are actually accelerating aging (181). If this is true, we should call *ad libitum* feeding “overfeeding,” an insight that has profound implications well beyond geroscience (211), and translating to humans the evidence from CR on animals will be as simple as to say: “avoid obesity”.

## Author Contributions

VS and AL wrote and revised the article. AL wrote Introduction and Conclusions. VS wrote all the other paragraphs. CS implemented and revised each section. All authors gave final approval of the submitted version.

### Conflict of Interest Statement

The authors declare that the research was conducted in the absence of any commercial or financial relationships that could be construed as a potential conflict of interest.

## Abbreviations

BMI: body mass index
CR: caloric restriction
ASC: Adipose-derived stem cells
UPR: unfolded protein response
HSC: hematopoietic stem cells
BM: bone marrow
BMAT: bone marrow adipose tissue
BM-MSC: bone marrow stromal stem cell
HFD: high fat diet
GH: growth hormone
IGF-1: insulin-like growth factor
IGFBP: IGF binding protein
HSP: heat shock protein
ER: endoplasmic reticulum
UPR: unfolded protein response.
